# Graphene-Based Nanomaterials in Soil: Ecotoxicity Assessment Using *Enchytraeus crypticus* Reduced Full Life Cycle

**DOI:** 10.3390/nano9060858

**Published:** 2019-06-05

**Authors:** Monique C. P. Mendonça, Natália P. Rodrigues, Marcelo B. de Jesus, Mónica J. B. Amorim

**Affiliations:** 1 Department of Biochemistry and Tissue Biology, Institute of Biology, University of Campinas, Campinas, São Paulo 13083-970, Brazil; dejesus@unicamp.br; 2 Department of Biology, CESAM, University of Aveiro, Aveiro 3810-193, Portugal; nataliapegorarorodrigues@gmail.com

**Keywords:** graphene oxide, reduced graphene oxide, terrestrial environment, survival, reproduction, hatching success

## Abstract

Graphene-based nanomaterials (GBNs) possess unique physicochemical properties, allowing a wide range of applications in physical, chemical, and biomedical fields. Although GBNs are broadly used, information about their adverse effects on ecosystem health, especially in the terrestrial environment, is limited. Therefore, this study aims to assess the toxicity of two commonly used derivatives of GBNs, graphene oxide (GO) and reduced graphene oxide (rGO), in the soil invertebrate *Enchytraeus crypticus* using a reduced full life cycle test. At higher exposure concentrations, GO induced high mortality and severe impairment in the reproduction rate, while rGO showed little adverse effect up to 1000 mg/kg. Collectively, our body of results suggests that the degree of oxidation of GO correlates with their toxic effects on *E. crypticus*, which argues against generalization on GBNs ecotoxicity. Identifying the key factors affecting the toxicity of GBNs, including ecotoxicity, is urgent for the design of safe GBNs for commercial purposes.

## 1. Introduction

Over the last decade, the production and use of nanomaterials (NMs) have been rapidly expanding due to a wide range of applications in many industrial sectors. Among them, graphene-based nanomaterials (GBNs) have attracted considerable interest because of their fascinating optical, electrical, mechanical, and thermal properties. These properties allowed the GBNs application in electronic devices, energy storage, biosensors, semiconductors, water purification (filter), biomedicine, tissue engineering, and drug/gene delivery [1,2]. From 2010 to 2017, the compound annual growth rate (CAGR) of graphene composites worldwide was estimated on 61% [3]. By 2023, the global market volume of graphene composites may reach 1521 tons [4].

Graphene nanomaterials consisting solely of carbon are known to be non-toxic; however, graphene derivatives like graphene oxide (GO) and reduced graphene oxide (rGO) can contain residual metals and impurities from the chemicals used in the treatment process for oxidation and reduction [5]. Moreover, GO and rGO can be functionalized with a wide variety of compounds, such as polymers and biomolecules, that can alter their structure and, consequently, their toxicity [6,7]. Thus, with the increasing use and production of GBNs, it is expected that large quantities of industrial waste will end up in the environment, representing a risk for human health and living organisms. Thus, systematic investigation of any potential toxic effects of GO/rGO is essential to ensure assessment of safety at biological and environmental levels.

The terrestrial environment is one of the primary sinks of NMs, and soil invertebrates are directly affected by these compounds. Hence, such organisms can serve as soil sentinels of quality disturbances, where several are used as surrogates, like enchytraeids (Enchytraeidae, Oligochaeta, Annelida). Enchytraeids are essential for organic matter decomposition and live in close contact with the soil pore water. Therefore, they are exposed to soil contamination via dermal, gastrointestinal, and respiratory routes, being often very sensitive to a broad range of stressors [8]. Furthermore, its use has been supported by the internationally accepted guidelines designed to assess the effects of substances on the survival and reproductive output of *Enchytraeus* sp. [9,10]. The evaluation of additional endpoints, e.g., hatching success, maturity, and growth, was proposed by Bicho et al. (2015) in a full life cycle test to complement the hazard assessment in different life cycle stages [11].

Currently, little is known about the impacts of GBNs on the terrestrial environment, and their effects on the life cycle of soil invertebrates remain largely unaddressed. Hence, we here evaluate the environmental impact of GBNs using *Enchytraeus crypticus.* The effects of the two commonly used derivatives of GBNs, GO, and rGO were investigated, using *Enchytraeus crypticus* in a reduced full life cycle test, measuring cocoons hatching and size, adult survival, and reproduction rate (number of juveniles).

## 2. Materials and Methods

### 2.1. Test Nanomaterials and Characterization

Commercial GO (catalog #763713, Sigma-Aldrich, St. Louis, MO, USA) were purchased in flakes and rGO (catalog #777684, Sigma-Aldrich) in powder. The GO flakes were suspended in deionized water (10 mg/mL) and sonicated in an ultrasonic bath for 30 min immediately prior to use. The characterization data of the commercial GBNs are available on the supplier’s webpage and have also been previously assessed [12,13]. Briefly, the tested GO was composed of carbon (42–52 wt %) and oxygen (≥36 wt %), and exhibited the size of 219 nm (performed using dynamic light scattering and zeta potential of −14.13 ± 11.1. Reduced GO was composed of carbon (≥75 wt %), nitrogen (>5 wt %) and oxygen (≤22 wt %), and exhibited a BET surface area of 450 m^2^/g. Further characterization of the size and zeta potential of rGO was not possible as it was added to the soil as a dry powder (not water-dispersed).

### 2.2. Test Organisms

Cultures of test species *Enchytraeus crypticus* (Enchytraeidae, Oligochaeta) were maintained in Petri dishes filled with agar, consisting of Bacti-Agar medium (Oxoid, Agar No. 1) and a mixture of salt solutions at the final concentrations of 2 mM CaCl_2_·2H_2_O, 1 mM MgSO_4_, 0.08 mM KCl, and 0.75 mM NaHCO_3_. The organisms were cultivated with a photoperiod of 16:8 h light/dark, temperature of 20 ± 1 °C, and fed autoclaved ground oats twice per week.

### 2.3. Test Soil and Spiking Procedure

The standard LUFA 2.2 natural soil (Speyer, Germany) was used. The composition is as follows: 1.77% organic matter, pH (0.01 M CaCl_2_) 5.5, 10.1 meq/100 g cation exchange capacity, 45.8% maximum water-holding capacity (WHC), grain size distribution of 7.3% clay (<0.002 mm), 13.8% silt (0.002 to 0.05 mm), and 78.9% sand (0.05 to 2.0 mm). Soil samples were oven-dried at 80 °C for 48 h before use. Spiking was performed according to the recommendations for nanomaterials by the Organisation for Economic Co-operation and Development (OECD) [14]. GO was added as aqueous solution to pre-moistened soil (25% (w/)w), whereas rGO was added as a dry powder to the soil as recommended for poorly dispersed materials. Since no toxicity data were available for soil invertebrates, GO and rGO were tested using a wide range of concentrations (0, 5, 250, and 1000 mg GO or rGO/kg dry soil.) Soil moisture was adjusted to 50% of the WHC and allowed to equilibrate for 24 h before starting exposure.

### 2.4. Exposure Experimental Design

An adapted version of the full life cycle test described in Bicho et al. (2015) was conducted [11]. Briefly, age-synchronized 1- to 2-day-old cocoons (n = 10 per replicate) were introduced in each test vessel containing 10 g of soil. Exposure period was 11 days (hatching success and size (area in mm^2^) endpoint) and 46 days (survival and reproduction endpoint) at a constant temperature of 20 ± 1 °C with a photoperiod of 16:8 h light/dark. Four replicates per treatment were used. After hatching, the test organisms were fed once a week with autoclaved ground oats, and the water loss was replenished.

**Figure 1:**
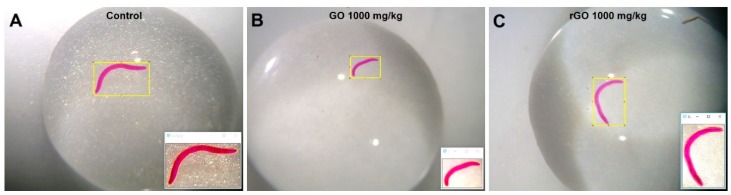
Illustration of the methodology used for quantitative analysis of *E. crypticus* size (in mm^2^). Representative images of (**A**) control, organisms exposed to either (**B**) 1000 mg graphene oxide (GO)/kg or (**C**) 1000 mg reduced graphene oxide (rGO)/kg. Insets show the same animals after contour delimitation using ImageJ software.

To extract organisms from the soil for counting, replicates were fixated with 96% ethanol and stained with 1% Bengal rose. After incubation overnight at 4 °C, soil samples were sieved in a 1.6, 0.5, and 0.3 mm mesh and the collected adults and juveniles were manually counted using a stereomicroscope (Zeiss Stemi 2000-C) to determine effects on survival and reproduction. The survival was determined on the adult survival, whereas reproduction was based on the number of juveniles produced. After counting, sizes were recorded for 11-day-old juveniles. The procedure consisted of placing each organism in a drop of water and photograph using a Dino-Eye camera and Dino-Lite software (Dino-Lite Digital Microscope, AnMo Electronics Corporation, New Taipei City, Taiwan). Size was measured by evaluating the area (mm^2^) from the object contour delimitation function using ImageJ software (NIH, Bethesda, MD, USA) after calibration, with the metric ruler, of the pixel/millimeter ratio (Figure 1).

### 2.5. Data Analysis

Results are presented as means ± standard error of the means (SEM) and analyzed for statistical significance by one-way analysis of variance (ANOVA), followed by Dunnett’s multiple comparison tests using GraphPad Prism 5.0 (GraphPad Software, San Diego, CA, USA). Student’s *t*-test (two-tailed, unpaired) was used to compare the treatment effects (GO vs. rGO). The effect of treatment and concentration, as well as their interaction, was further determined using two-way ANOVA. *p* value < 0.05 was considered for statistical significance. Effect concentrations (ECx) were estimated using the Toxicity Relationship Analysis Program (TRAP 1.30a) applying the best-fitting regression model (logistic 2 parameters or threshold sigmoid 2 parameters).

## 3. Results

**Figure 2:**
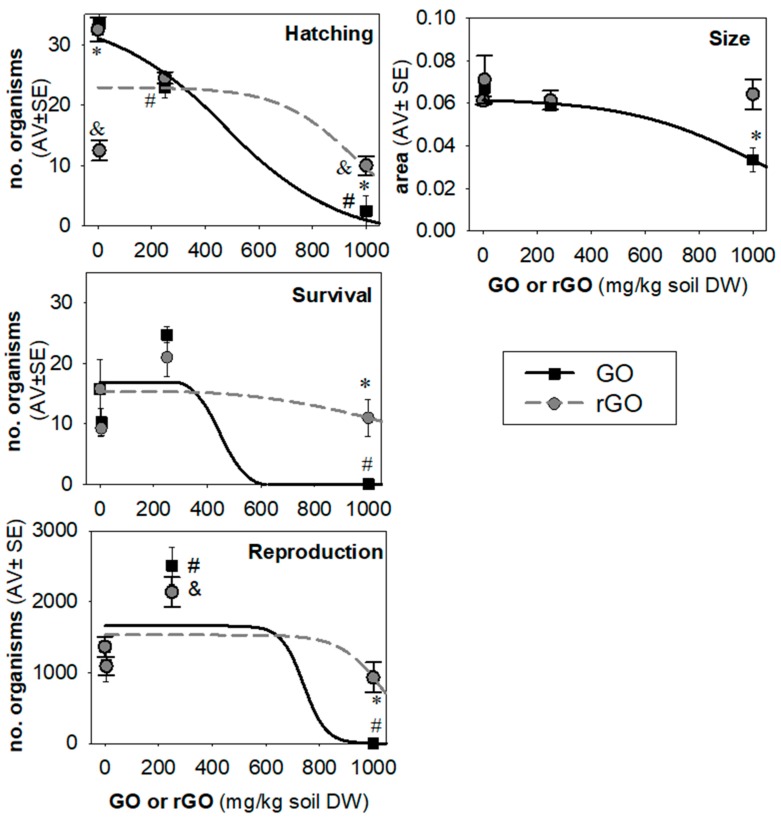
Effects of graphene oxide (GO) and reduced graphene oxide (rGO) in *Enchytraeus crypticus* after 11 days (hatching and size (area in mm^2^) and 46 days of exposure (reproduction and survival). All values are expressed in average ± standard error of the means (AV ± SEM). Lines represent the data fit to model. #: *p* < 0.05 GO vs. 0 (control group); & *p* < 0.05 rGO vs. 0 (control group) following one-way ANOVA plus Dunnett’s post hoc test. *: *p* < 0.05 GO vs. rGO compared to the corresponding concentration following Student’s *t*-test.

No significant changes occurred in the soil pH (5.7 ± 0.06) during the experimental period regardless of the treatment condition. Results of the exposure of the soil invertebrate *E. crypticus* to GBNs (GO and rGO) using the reduced full life cycle test (cocoon hatching, size, survival, and reproduction) can be observed in Figure 2.

The dose response effect concentrations were estimated (Table 1) and predictive models and parameters are given.

Exposure to GO caused a clear dose–response effect on hatching success. Significant decrease upon hatching was observed at 250 mg GO/kg (*p* < 0.05), and even higher at 1000 mg GO/kg (*p* < 0.001). For rGO-exposed organisms, hatching was significantly lower in cocoons treated with the lowest concentration of 5 mg rGO/kg (*p* < 0.001) similar to the observed in the highest concentration of 1000 mg rGO/kg (*p* < 0.05). Comparing GO and rGO, we observe differences in the hatchability of cocoons at the concentrations of 5 and 1000 mg/kg. Interestingly, in terms of size of the hatched organisms, it decreased only for GO exposure (1000 mg GO/kg, *p* < 0.05).

Survival and reproduction were significantly affected by GO at the highest exposure concentration tested (*p* < 0.001), showing 100% mortality. On the other hand, 1000 mg rGO/kg showed little to no toxicity. Comparing GO and rGO, GO was significantly more toxic towards *E. crypticus* as shown by the decrease in the survival (*p* < 0.05) and reproduction rate (*p* < 0.001).

Additionally, two-way ANOVA was conducted to compare differences between GO and rGO treatment on different concentrations and the interaction between the variables (treatment and concentration) (Table S1). The analysis revealed that treatment significantly affected the reproduction (*p* < 0.01), while the concentration affected all life cycle parameters (hatching, survival, and reproduction, *p* < 0.0001), except for the size (*p* = 0.0896). There was an interaction between treatment and concentration in relation to hatching (*p* < 0.001) and reproduction (*p* < 0.01).

## 4. Discussion

Comparison between GO and rGO showed higher toxicity of GO, which could be associated with higher oxidative stress, given their distinct structural and functional surface properties [15]. Graphene oxide is a single-atomic-layered material comprising carbon, hydrogen, and oxygen molecules derived from the exfoliation of graphite oxide. The oxidation of graphite resulted in abundant oxygenated functional groups, such as hydroxyl and epoxides on the basal plane and carbonyl and carboxyl groups at the edges. Subsequent reduction of GO is efficient to remove some of these oxygen-containing functional groups and recovery the π-conjugated structure of graphene, leading to the formation of rGO [1]. In general, graphene materials, which contain a higher density of functional groups, have a higher chance of interacting with cells, resulting in cell deposition and increased cytotoxicity. Thus, it suggests that the higher oxygen content of GO was responsible for the increased toxicity observed compared to their reduced counterpart (rGO) [16,17,18,19].

Similarly to *E. crypticus*, in other species, e.g., in the nematode *Caenorhabditis elegans*, the reproductive toxicity from GO was higher compared to rGO [20]. In *E. crypticus*, the higher toxicity of GO compared to rGO was also preceded by a higher size reduction of the hatched juveniles. This could well mean a correlation between size of offspring and survival levels. The relationship between organisms size and performance have been reported before (e.g., [21,22,23]) and is often linked to energy allocation and trade-offs, e.g., fewer and larger animals are more fit to stress than many and smaller (K and R strategies). This was not the case here, where we observed a lower number of also smaller hatched juveniles. Effects seem additive and, hence, severely decreased survival occurred.

More of a physiological aspect of the organisms, the effects observed in terms of hatching success (11 days) were in good agreement with effects observed in survival (46 days), for both GO and rGO, indicating a good predictability of effects between early and later stages of development. This is not always the case but it has been observed before for *E. crypticus*, e.g., when exposed to silver nitrate and silver nanoparticles [24]. On the other hand, exposure to copper oxide nanomaterials [25] or nickel nanoparticles [26] showed that the observed decreased hatching was in fact a delay, and organisms recovered with time. Similarly, the observed reduction in hatching at 5 and 1000 mg rGO/kg was partly a delay and effects were diluted with time, as observed in terms of survival and reproduction.

The 250 mg GO or rGO/kg seems to cause a hormesis-like effect, i.e., a *stimulus* in the performance of the organisms at low doses, as observed by an increase in both survival and reproduction. This has also been observed when *E. crypticus* were exposed to multiwalled carbon nanotubes [27], indicating that this could be the result of beneficial added carbon to the system, up to a certain maximum level.

The survival and reproduction followed a similar response pattern and, hence, effects on reproduction appear to be a direct consequence of decreased survival rather than a specific reproductive impairment. In *C. elegans*, and as opposed to this, Kim et al. (2018) suggested that GO exposure caused reproductive toxicity by suppressing spermatogenesis of *C. elegans* hermaphrodites during development, resulting in decreased sperm numbers and progeny numbers [28].

Previous studies showed that GO is toxic to several organisms, including microbial communities, bacteria, fungi, plants, tadpoles, and rodents [18,29,30,31,32]. It has been shown that the presence of GO (50 to 300 mg/L) can significantly impact bacterial metabolic activity, bacterial viability, and biological removal of nutrients, such as organics, nitrogen, and phosphorus, in the activated sludge. These effects lead to compromised wastewater treatment performance since an efficient biological wastewater treatment requires the functioning of diverse microbial species [33]. Conversely, Esquivel-Gaon and co-workers did not observe any changes on bacterial growth, morphology, and DNA fragmentation of the common soil bacteria *Pseudomonas putida* and on nitrifying bacteria after rGO exposure [34].

In invertebrates, GO was reported to decrease the survival rate and inhibit the swimming behavior of the crustacean *Amphibalanus amphitrite* nauplii, to reduce the burrowing activity of the oligochaete *Tubifex tubifex*, and to induce cellular damage and reduce the metabolic capacity (higher glycogen content) of the polychaeta *Diopatra neapolitana* [35]. Results from other studies also indicate that the reduced form of GO caused higher toxicity compared to the oxidized form [35,36,37,38]. Although most of the studies attributed these differences between GO and rGO to the differences in experimental approach, type base of material, size, thickness (number of layers), shape, and coatings, it is also necessary to consider the oxygen content and the carbon radical density of the GBN surfaces [18,19].

The potential environmental risk related to these GBN compounds are not yet clear enough and further mechanistic studies are recommended as it is very important to ensure the environmental safety of GBNs.

## 5. Conclusions

Chronic exposure to GO significantly impaired survival and reproduction (rGO caused nearly no toxicity up to 1000 mg/kg). The differences observed indicated that the higher oxygen content of GO must play a major role in the induction of toxicity towards *E. crypticus*. Hormesis at lower doses (250 mg/kg) was observed and could be due to the beneficial added carbon to the system. Effects of GO occurred at early life stage development with decreased hatching success (and smaller size of hatched organisms). There was good predictability of GO effects between early and later stages of development, i.e., hatching and survival. The use of a full life cycle test allowed further understanding of the mechanisms of action of GO. Further in-depth studies are recommended including the usage of more species and endpoints.

## Figures and Tables

**Table 1:** Estimated effect concentrations (EC) for *Enchytraeus crypticus* when exposed to GO and rGO in a full life cycle test, in terms of hatching and size (area, in mm^2^) at day 11, survival, and reproduction at day 46. Logistic 2 parameters and threshold sigmoid 2 parameters models were used. Confidence intervals (95% CI) are shown in brackets. n.e.: no effect; n.d.: not determined/out of range; S: Slope; Y0: interception.

Nanomaterial/Endpoint	EC_10_ (mg/kg)	EC_20_ (mg/kg)	EC_50_ (mg/kg)	EC_80_ (mg/kg)	Model and Parameters
**Hatching**					
GO	129 (−36–294)	202 (123–282)	329 (197–460)	455 (159–751)	Log 2 par S: 0.0009 Y0: 33; R^2^ = 0.9
rGO	753 (n.d.)	834 (n.d.)	973 (n.d.)	1111 (n.d.)	Log 2 par S: 0.0008; Y0: 23.2; R^2^ = 0.3
**Size**					
GO	490 (82–898)	685 (385–984)	1017 (725–1310)	1349 (858–1841)	Log 2 par S: 0.00092; Y0: 0.06; R^2^ = 0.2
rGO	n.e.	n.e.	n.e.	n.e.	-
**Reproduction**					
GO	650 (n.d.)	683 (n.d.)	740 (n.d.)	798 (n.d.)	Log 2 par S: 0.006; Y0: 1658; R^2^ = 0.6
rGO	860 (n.d.)	925 (n.d.)	1034 (n.d.)	1144 (n.d.)	Log 2 par S: 0.003; Y0: 1530; R^2^ = 0.2
**Survival**					
GO	353 (n.d.)	384 (n.d.)	447 (n.d.)	492 (n.d.)	Thresh 2 par S: 0.002; Y0: 16.9; R^2^ = 0.5
rGO	696 (n.d.)	881 (n.d.)	1248 (n.d.)	1512 (n.d.)	Thresh 2 par S: 0.00099; Y0: 15.3; R^2^ = 0.7

## Supplementary Materials

The following are available online at https://www.mdpi.com/2079-4991/9/6/858/s1, Table S1: Results of two-way analysis of variance of the effects of GO and rGO on life cycle parameters.
