# Supplementary material for: Graphene-Based Nanomaterials in Soil: Ecotoxicity Assessment Using Enchytraeus crypticus Reduced Full Life Cycle
Source: Nanomaterials (Basel). 2019 Jun 5;9(6):858. doi: 10.3390/nano9060858 (PMC6631203; doi:10.3390/nano9060858)
Supplement: Supplementary file 1 [file nanomaterials-09-00858-s001.pdf]

Supplementary Information

Graphene-Based Nanomaterials in Soil: Ecotoxicity Assessment Using *Enchytraeus crypticus* Reduced Full Life Cycle

Monique C. P. Mendonça [1,2,*,†], Natália P. Rodrigues [2,†], Marcelo B. de Jesus [1] and Mónica J. B. Amorim [2,*]

[1] Department of Biochemistry and Tissue Biology, Institute of Biology, University of Campinas, Campinas, São Paulo 13083-970, Brazil; dejesus@unicamp.br

[2] Department of Biology, CESAM, University of Aveiro, Aveiro 3810-193, Portugal; nataliapegorarorodrigues@gmail.com

* Correspondence: m115623@dac.unicamp.br (M.C.P.M.); mjamorim@ua.pt (M.J.B.A.)

† These authors contributed equally to this work.

Table S1. Results of two-way analysis of variance of the effects of GO and rGO on life cycle parameters.

**Parameter**	**Effect**	**P**	**F**
Hatching	Treatment	0.0600	3.86
	Concentration	p < 0.0001	55.07
	Interaction	p < 0.0001	16.17
Size	Treatment	0.0932	2.83
	Concentration	0.0896	2.18
	Interaction	0.3037	1.21
Survival	Treatment	0.4793	0.516
	Concentration	p < 0.0001	1.06
	Interaction	0,1111	2.23
Reproduction	Treatment	0.0041	10.06
	Concentration	p < 0.0001	11.28
	Interaction	0.0026	6.318
